# Exploration and Practice of the First Clinical Medical Postdoctoral Program in China: Retrospective, Nonrandomized, Controlled Study

**DOI:** 10.2196/65622

**Published:** 2025-11-24

**Authors:** Lingda Zhang, Lianghong Sun, Honglei Li

**Affiliations:** 1 Department of Education, The Second Affiliated Hospital, Zhejiang University School of Medicine, 88 Jiefang Rd, Hangzhou, 310009, China, 86 057187784594

**Keywords:** clinical medicine postdoctoral program, standardized residents training, clinical competence, scientific research ability, teaching skills, comprehensive ability

## Abstract

**Background:**

To further optimize the clinical and scientific training of high-level doctoral graduates, the Office of the National Postdoctoral Administration launched a clinical postdoctoral program in 2015. This program provides postdoctoral clinical medicine trainees with 3 years of individualized, intensive training through a full mentorship system, interdisciplinary collaboration, and a multiteam teaching platform.

**Objective:**

This study aimed to compare the effectiveness of this novel clinical postdoctoral training program against conventional doctoral training, using specific, quantifiable metrics. Our primary research questions were as follows: (1) Does the program lead to superior clinical performance, as measured by theoretical examination scores and Case Mix Index (CMI)? (2) Does it enhance scientific research productivity, measured by publication output and success rates in procuring provincial and national NSFC (National Natural Science Foundation of China) funds? (3) Are there differences in teaching capacity and overall career advancement?

**Methods:**

This was a retrospective, nonrandomized controlled study. Doctoral graduates who entered the hospital for standardized residency training between 2015 and 2019 were enrolled and divided into a postdoctoral training group (n=23) and a doctoral training group (n=106).

**Results:**

The postdoctoral group demonstrated significantly higher clinical performance, as indicated by higher theoretical examination scores (445.70, SD 14.67 vs 435.12, SD 15.29; *P*=.003) and a higher median CMI (1.14 vs 0.92, *P*=.03), reflecting greater ability to manage complex clinical cases. In terms of research productivity, the postdoctoral group outperformed the doctoral group in the number of published papers (2.35, SD 2.39 vs 1.11, SD 1.47; *P*=.002) and the proportion of approved provincial-level NSFC projects (47.83% vs 17.00%, *P*=.001). However, no significant differences were observed in the acquisition of national-level NSFC funding (60.87% vs 44.34%, *P*=.15), teaching capacity (0.22, SD 0.518 vs 0.10, SD 0.306; *P*=.16), or overall competency indicators such as the rate of professional title promotion (100% vs 94.34%, *P*=.53) and the attainment of a master's-degree supervisor qualification (13.04% vs 5.67%, *P*=.42).

**Conclusions:**

The clinical postdoctoral training program demonstrates promising effectiveness in enhancing both clinical performance and scientific innovation among medical trainees. These findings support the value of integrating clinical practice, research, and mentorship in advanced postgraduate medical education and suggest that this model is worth promoting in more medical institutions.

## Introduction

### Background

China’s medical education system has undergone significant reform in recent years, evolving into a 3-stage model: medical school education, standardized residency training (SRT), and continuing professional development. SRT serves as the cornerstone of clinical training, aiming to produce physicians who are competent in managing common diseases with strong ethical standards and clinical skills [1-3]. However, while SRT focuses on standardized clinical practice, it often falls short in cultivating research literacy, academic leadership, and innovation capabilities—key competencies required for high-level clinical scholars.

To address this gap, the Office of the National Postdoctoral Management Committee launched a clinical postdoctoral training program in 2015 [4]. As the first pilot institution, Zhejiang University introduced a novel training model that builds upon SRT but goes beyond it by integrating advanced clinical training with rigorous scientific research and teaching development [5].

This program is specifically designed for highly qualified medical doctors who have completed their doctoral studies and seek to become leaders in both clinical practice and biomedical research. Unlike traditional SRT, which emphasizes competency in routine diagnosis and treatment, the clinical postdoctoral program enhances trainees’ abilities in clinical reasoning and management of complex cases, original research and evidence-based medicine, medical education and mentorship, and innovation and translational application of clinical findings [6].

The curriculum combines structured clinical rotations with independent research projects under the supervision of multidisciplinary mentors. It also includes formal teaching experiences, international exchanges, and interdisciplinary collaboration, all aimed at fostering a new generation of clinicians who can lead in academia, contribute to global health, and drive clinical innovation.

Ultimately, the program seeks to cultivate clinician scientists and clinician educators who possess not only excellent clinical expertise but also the capacity to conduct high-impact research, mentor future generations of physicians, and translate scientific discoveries into clinical practice. This represents a significant departure from conventional postgraduate medical training models seen worldwide and aligns more closely with integrated clinical-academic pathways found in some elite programs in North America and Europe [7-9].

This study focuses on the pioneering program at Zhejiang University to evaluate its effectiveness in bridging the gap between clinical training and academic development, thereby providing evidence for its potential role in advancing China’s medical education system.

### Objectives

While Zhejiang University School of Medicine and its Second Affiliated Hospital have a long-standing tradition of excellence in medical education, traditional postresidency training often fails to provide integrated development in advanced clinical practice, research, and teaching. To address this, we launched the Clinical Medical Postdoctoral Training Program in 2015. This study aims to evaluate the effectiveness of this program’s first 8 years of implementation (2015‐2023) through a retrospective, nonrandomized, controlled design.

Specifically, we sought to compare the postdoctoral trainees with conventionally trained counterparts using a set of predefined, quantifiable metrics as proxies for key competencies: (1) clinical performance measured based on standardized theoretical examination scores and the Case Mix Index (CMI) of treated patients, (2) research productivity measured by publication output and success rates in obtaining provincial and national-level NSFC (National Natural Science Foundation of China) funds, (3) teaching engagement measured by the number of formal teaching sessions conducted, and (4) career advancement measured by the rate of professional title promotion and qualification as a master’s-degree supervisor.

We hypothesized that this program would significantly improve participants’ clinical reasoning, research productivity, and teaching capabilities compared to conventional postresidency pathways. The findings are expected to be of particular interest to medical educators, hospital administrators, and policymakers involved in designing advanced clinical training programs, especially in countries seeking to strengthen their clinical academic workforce.

## Methods

### Ethical Considerations

This study was approved by the Ethics Committee of The Second Affiliated Hospital, Zhejiang University School of Medicine (approval number 2023-0974). Informed consent was waived by our Institutional Review Board because of the retrospective nature of our study.

### Study Design and Participants

We conducted a retrospective, nonrandomized, controlled study. The study population consisted of medical doctoral graduates who entered the hospital between 2015 and 2019 and were required to undergo SRT. Individuals without a numeric score on the SRT completion test were excluded to ensure the quantitative evaluation of training outcomes.

Participants were divided into 2 groups based on their training pathway:

- Doctoral group (control): graduates who completed only SRT.
- Postdoctoral group (intervention): graduates who successfully applied for and completed the additional 3-year Clinical Medicine Postdoctoral Training Program.

Admission to the postdoctoral program was competitive, based on academic merit, clinical performance, and personal motivation. The program provided enhanced, structured training in clinical practice, scientific research, and teaching under a dedicated mentorship team [10,11].

### Outcome Measures

To evaluate the program’s effectiveness, we selected a comprehensive set of quantitative metrics designed to proxy competencies in clinical practice, scientific research, teaching, and career advancement (Table 1). Data for all metrics were collected over the 3-year training period. All metrics were analyzed with stratification by age, gender, clinical discipline, and prior training duration.

**Table 1.** Primary outcome measures and their operational definitions.

Metric category	Operational definition and data source	Construct measured
Clinical performance		
Theoretical examination score	Final examination score; source: Education Department	Mastery of clinical knowledge
SRT^a^ completion pass rate	Binary outcome (pass/fail) for the standardized national residency completion examination; source: Education Department	Attainment of the minimum competency standard required for independent clinical practice
Annual assessment score	Composite annual review score; source: Education Department	Overall clinical performance and professionalism
Case Mix Index	The average diagnosis-related group (DRG) weight of patients treated; source: Medical Record Room	Complexity of clinical experience
Scientific research ability		
Number of papers published	Total count of peer-reviewed research articles published; source: Scientific Research Department	Scientific output and research ability
The number of achievement awards won as a participant	The number of scientific research achievement awards (at the provincial/ministerial level or above) won by the trainee as a contributor; source: Scientific Research Department	Peer recognition and impact of research output
National/provincial NSFC^b^ projects	Total number of grants obtained as a principal investigator; source: Scientific Research Department	Competitiveness in securing research funding
Teaching ability		
Teaching engagement	Total number of teaching articles/teaching reform projects/teaching competitions; source: Education Department	Contribution to medical education
Comprehensive ability		
Professional title promotion	Binary outcome (yes/no) for promotion from a junior to an intermediate title; source: Human Resources	Institutional recognition of competence
Master’s degree supervisor qualification	Binary outcome (yes/no) for being approved as a supervisor; source: Education Department	Recognition of academic standing

a SRT: standardized residency training.

b NSFC: National Natural Science Foundation of China.

### Data Collection and Processing

Data were collected from the hospital’s departmental databases (Education Department, Human Resources, Scientific Research Department, and Medical Record Room). The collection process involved extracting structured data from electronic source files, including examination records, personnel files, grant management systems, and publication databases. To ensure data quality, we handled missing data through listwise deletion and checking for implausible values or outliers. This process was conducted by 2 independent researchers to minimize error.

### Statistical Analysis

SPSS (version 27.0; IBM Corp) was used for statistical evaluation of the data. Numerical/count data were expressed as the n (%) values, and the *t *test or chi-square test was used for comparison between groups. Normally distributed data are expressed as mean (SD) values, and the *t* test was used for comparison between groups. Nonnormally distributed data are expressed as median (IQR) values, and between-group comparisons were made using the Mann-Whitney *U* test. *P* values of <.05 were considered statistically significant.

## Results

### Study Participants

**Figure 1.**
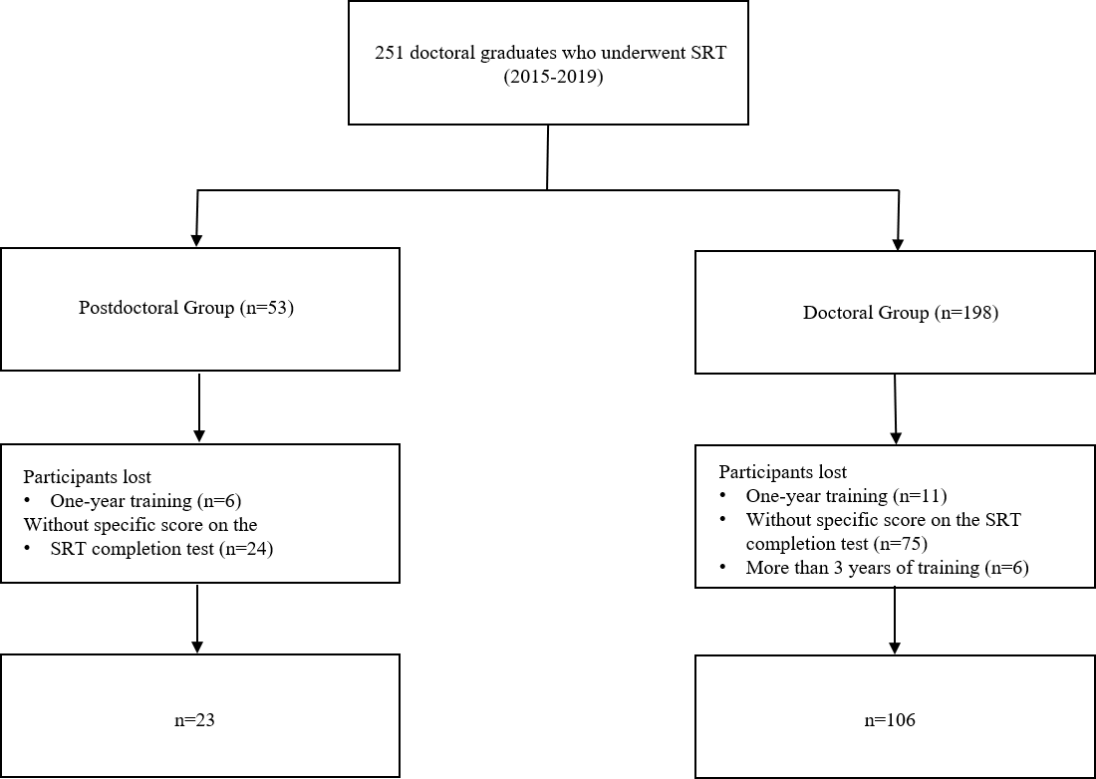
Flowchart for the study. A total of 251 trainees (admitted from 2015 to 2019) met the inclusion criteria of this study. Exclusion criteria: 1 year of training, more than 3 years of training, and not having a specific score on the SRT completion test. In total, 129 trainees were finally enrolled, including 23 postdoctoral trainees and 106 control trainees. SRT: standardized residency training.

A total of 251 trainees (admitted from 2015‐2019) met the inclusion criteria of this study. After excluding 17 trainees with less than 1 year of training, 6 trainees with more than 3 years of training, and 99 trainees without a specific score on the SRT completion test, 129 trainees were finally enrolled, including 23 postdoctoral trainees and 106 control trainees (Figure 1). Table 2 shows that there was no statistically significant difference between the two groups of trainees in terms of gender, age, distribution of disciplines, and other aspects. The difference in training years was statistically significant (*P*<.001).

**Table 2.** General information of all the trainees enrolled in the study (N=129).

Parameter	Doctoral (n=106)	Postdoctoral (n=23)	Statistic (*df*)	*P* value
Gender, n (%)			0.706 (1)^a^	.40
Male	59 (55.66)	15 (65.22)
Female	47 (44.34)	8 (34.78)
Age (years), mean (SD)	28.05±1.73	27.85±1.91	0.485 (127)^b^	.63
Discipline, n (%)			1.346 (2)^a^	.51
Internal medicine	31 (29.25)	4 (17.39)
Surgery	36 (33.96)	9 (39.13)
Others	39 (36.79)	10 (43.48)
Training years, n (%)			18.612 (1)^a^	<.001
2 years	16 (15.09)	13 (56.52)
3 years	90 (84.91)	10 (43.48)

a Chi-square values.

b *t* values.

### Clinical Performance

The postdoctoral group demonstrated significantly higher clinical performance, as measured based on the standardized theoretical examination scores (445.70, SD 14.67 vs 435.12, SD 15.29; *P*=.003). The CMI was also significantly higher in the postdoctoral group (1.14 vs 0.92, *P*=.03), indicating exposure to and management of more complex clinical cases. However, analysis revealed no statistically significant differences between the 2 groups regarding the three key metrics: graduation examination pass rate, annual theoretical assessment scores, and annual skill assessment scores ( as shown in Table 3).

**Table 3.** Comparison between the doctoral and postdoctoral groups.

Parameter	Doctoral (n=106)	Postdoctoral (n=23)	Statistic (*df*)	*P* value
Clinical performance				
Theoretical scores of SRT^a^ completion test, mean (SD)	435.12 (15.29)	445.70 (14.76)	–3.024 (127)^b^	.003
Pass rate of graduation examination, n (%)	99 (93.40)	20 (86.96)	0.380 (1)^c^	.54
Annual assessment of theoretical scores, mean (SD)	81.84 (10.44)	80.70 (12.67)	0.455 (127)^b^	.65
Annual assessment of skill scores, mean (SD)	86.24 (4.81)	87.85 (4.72)	–1.455 (127)^b^	.15
CMI^d^, median (IQR)	0.92 (0.39)	1.14 (0.46)	–2.130^e^	.03
Scientific research ability				
Number of papers published, mean (SD)	1.11 (1.47)	2.35 (2.39)	–3.225 (127)^b^	.002
Number of achievement awards won as a participant, mean (SD)	0.06 (0.303)	0.09 (0.288)	–0.439 (127)^b^	.66
Proportion of national NSFC^f^ approvals, n (%)	47 (44.34)	14 (60.87)	2.072 (1)^c^	.15
Number of provincial NSFC applications approved, n (%)	18 (17.00)	11 (47.83)	10.318 (1)^c^	.001
Teaching ability				
Teaching articles/teaching reform projects/teaching competitions, mean (SD)	0.10 (0.306)	0.22 (0.518)	–1.402 (127)^b^	.16
Comprehensive ability, n (%)				
Promotion of professional title	100 (94.34)	23 (100)	0.387 (1)^c^	.53
Master’s degree supervisor	6 (5.67)	3 (13.04)	0.654 (1)^c^	.42

a SRT: standardized residency training.

b *t* value.

c Chi-square value.

d CMI: Case Mix Index.

e Mann-Whitney *U* test value; df value not applicable.

f NSFC: National Natural Science Foundation of China.

### Scientific Research Ability

According to the statistics provided by the Scientific Research Department of the hospital, the number of published papers per capita was significantly higher in the postdoctoral group than in the doctoral group (2.35, SD 2.39 vs 1.11, SD 1.47; P=.002). In the doctoral group, 18 of 106 (17.00%) participants received a provincial NSFC fund, while in the postdoctoral group, 11 of 23 (47.83%) participants received a provincial NSFC fund, and the difference was significant between the 2 groups (*P*=.001). In terms of the number of provincial NSFC funds and the number of published papers per capita, the postdoctoral group had significantly better metrics than the doctoral group. As for NSFC applications, 14 of 23 (60.87%) postdoctoral participants and 47 of 106 (44.34%) doctoral trainees received approval. Although there was no significant difference between the 2 groups, the postdoctoral group may have been granted more funding if the sample size was large enough.

### Teaching Skills

In this study, the trainees’ teaching ability was described by the number of approved teaching improvement projects, number of published teaching papers, and whether they had participated in teaching competitions and awards. These indicators allow for the assessment of the trainees’ participation and creativity in clinical teaching. As most trainees did not fully participate in clinical teaching and few papers were published or approved, there was no significant difference between the 2 groups.

### Comprehensive Ability

In this study, there was no significant difference between the 2 groups on comparing whether or not an intermediate title was obtained within 3 years. On the other hand, trainees are eligible to become graduate supervisors, which requires a strong scientific research background. Applicants must meet the following criteria: they must chair at least 1 ongoing national or provincial research project, publish academic papers in high-level journals as first author or corresponding author, or receive a research award at the provincial or ministerial level or higher. A certified supervisor's evaluation is additionally necessary. Due to the very limited number of supervisors authorized to supervise graduate trainees, there was no significant difference between the postdoctoral and doctoral groups.

## Discussion

### Principal Findings

In this study, we compared the competencies of medical graduates who completed SRT (the doctoral group) with those who undertook the advanced clinical postdoctoral program (the postdoctoral group). The principal findings reveal distinct advantages for the postdoctoral group in key areas. Specifically, these trainees demonstrated a superior capacity to manage complex clinical cases, as evidenced by a significantly higher median CMI (1.14 vs 0.92, *P*=.03). Their research productivity was also markedly greater, reflected in a higher number of published papers (2.35, SD 2.39 vs 1.11, SD 1.47; *P*=.002) and a significantly greater success rate in securing provincial-level NSFC-funded projects (47.83% vs 17.00%, *P*=.001). Furthermore, the postdoctoral group achieved significantly higher scores on standardized theoretical examinations over the final 3 years of the study period (445.70, SD 14.67 vs 435.12, SD 15.29; *P*=.003), indicating a deeper mastery of medical knowledge. Collectively, these quantitative findings support the hypothesis that the integrated, postdoctoral training model more effectively enhances clinical readiness, scientific innovation, and theoretical understanding among medical graduates.

### Implications of Findings

The findings from this study have significant implications for advancing medical education and workforce development. The superior clinical performance of the postdoctoral group, as quantified by higher theoretical examination scores and a greater ability to manage complex cases (reflected in the significantly higher CMI), suggests that such integrated training can produce clinicians with enhanced diagnostic acumen and decision-making confidence, which may directly translate to improved patient care and clinical outcomes. Furthermore, the program’s effectiveness in cultivating physician scientists is clearly demonstrated by the marked increase in research output and success in securing provincial-level grants, bridging a critical gap between clinical practice and scientific innovation. While teaching competence showed limited quantitative difference, the structured mentorship and teaching experiences are posited to foster essential communication skills and educational confidence, building a foundation for future academic leaders. Collectively, these results provide a strong evidence-based rationale for other institutions and policymakers to adopt similar integrated clinical academic training models, underscoring the value of a unified framework that concurrently develops clinical, research, and teaching competencies to prepare physicians for multifaceted roles in modern health care.

### Comparison to the Literature

Our findings align with those of previous studies highlighting the importance of structured postdoctoral training in fostering clinical and academic excellence [6,10-12]. For instance, integrated clinical academic training models in Western countries—such as clinical fellowships and combined MD/PhD programs—have been shown to enhance research output and leadership roles among physicians [13,14].

However, unlike traditional postresidency research fellowships, the clinical postdoctoral program described here places equal emphasis on clinical service, academic inquiry, and teaching, reflecting a unique model tailored to China’s evolving health care and educational systems. Our results contribute to the growing body of evidence supporting innovative postdoctoral medical training models that extend beyond conventional residency programs.

### Strengths and Limitations

This study represents one of the first efforts to evaluate the effectiveness of a clinical postdoctoral training program in mainland China, offering a comprehensive assessment of trainees’ clinical, research, and teaching competencies. A key strength lies in the use of objective performance indicators, such as the CMI and research output metrics, which provide quantifiable evidence of clinical complexity management and academic productivity. Additionally, the inclusion of multiple competency domains allows for a more holistic understanding of the program’s impact on professional development. However, several limitations should be acknowledged. As a single-center study with a relatively small sample size (23 postdoctoral participants), the findings may lack generalizability and statistical power, increasing the risk of type II error. It should be noted that this analysis only included participants who completed the entire program and possessed complete assessment data. Exclusions from the final analysis were primarily for 2 reasons: voluntary withdrawal due to personal circumstances and the absence of a quantitative theoretical score in the completion examination, which precluded a fair comparative assessment. These exclusions may introduce elements of attrition and selection bias. The 3-year observation period may also be insufficient to capture the long-term developmental trajectory of medical professionals. Furthermore, selection bias cannot be ruled out, as participation in the postdoctoral program was self-selected, potentially reflecting preexisting differences in motivation or academic background. Some outcome measures, particularly those related to teaching and comprehensive abilities, relied on qualitative or semiquantitative assessments, limiting their objectivity and reproducibility. To address these limitations, future studies should adopt multicenter designs with larger cohorts and extended follow-up periods to better assess the longitudinal impact of the program. In addition, more standardized and validated tools should be developed to objectively measure teaching and comprehensive competencies. Comparative analyses with international postgraduate training models could further inform best practices and opportunities for cross-learning in clinician scientist development.

### Conclusion

As a new medical education mode in China, the clinical medical postdoctoral training program enhances the trainees’ clinical performance and scientific research innovation ability, which is helpful to cultivate the top innovative talents in clinical medicine. This training mode is effective and can be promoted in various medical teaching institutions as an important supplement to the current medical degree system in China.

## Abbreviations

CMI: Case Mix Index
NSFC: National Natural Science Foundation of China
SRT: Standardized Residents Training
